# Lung Transplantation in a New Era in the Field of Cystic Fibrosis

**DOI:** 10.3390/life13071600

**Published:** 2023-07-21

**Authors:** Wei Huang, Alexander T. Smith, Maksim Korotun, Aldo Iacono, Janice Wang

**Affiliations:** 1 Department of Medicine, Donald and Barbara Zucker School of Medicine at Hofstra/Northwell, Hempstead, NY 11549, USA; whuang6@northwell.edu (W.H.); mkorotun@northwell.edu (M.K.); aiocono1@northwell.edu (A.I.); jwang@northwell.edu (J.W.); 2 Division of Pulmonary, Critical Care, and Sleep Medicine, Department of Medicine, Donald and Barbara Zucker School of Medicine at Hofstra/Northwell, Hempstead, NY 11549, USA; 3 Institute of Health System Science, Feinstein Institute for Medical Research, Northwell Health, Manhasset, NY 11030, USA

**Keywords:** cystic fibrosis, lung allocation, lung transplantation, CFTR modulator therapy

## Abstract

Lung transplantation for people with cystic fibrosis (PwCF) is a critical therapeutic option, in a disease without a cure to this day, and its overall success in this population is evident. The medical advancements in knowledge, treatment, and clinical care in the field of cystic fibrosis (CF) rapidly expanded and improved over the last several decades, starting from early pathology reports of CF organ involvement in 1938, to the identification of the CF gene in 1989. Lung transplantation for CF has been performed since 1983, and CF now accounts for about 17% of pre-transplantation diagnoses in lung transplantation recipients. Cystic fibrosis transmembrane conductance regulator (CFTR) modulators have been the latest new therapeutic modality addressing the underlying CF protein defect with the first modulator, ivacaftor, approved in 2012. Fast forward to today, and we now have a growing CF population. More than half of PwCF are now adults, and younger patients face a better life expectancy than they ever did before. Unfortunately, CFTR modulator therapy is not effective in all patients, and efficacy varies among patients; it is not a cure, and CF remains a progressive disease that leads predominantly to respiratory failure. Lung transplantation remains a lifesaving treatment for this disease. Here, we reviewed the current knowledge of lung transplantation in PwCF, the challenges associated with its implementation, and the ongoing changes to the field as we enter a new era in the care of PwCF. Improved life expectancy in PwCF will surely influence the role of transplantation in patient care and may even lead to a change in the demographics of which people benefit most from transplantation.

## 1. Background

Cystic fibrosis (CF) is an autosomal recessive disease that results from inheriting two copies of a pathogenic mutation in the cystic fibrosis transmembrane conductance regulator (CFTR) gene, one from each parent [1]. The encoded CFTR protein is a cyclic adenosine monophosphate (cAMP)/protein kinase-regulated apical membrane anion channel expressed throughout numerous cells of the body, ranging from those of the lungs, intestines, kidneys, and pancreas [2] to lymphocytes and fibroblasts [3], and to cells of the central and peripheral nervous system [4]. Its most well-defined function is in epithelial cells where it typically facilitates flow of chloride ions across the cell membrane and allows for preservation of the balance between salt and water on several mucosal surfaces of the body [5]. CFTR total activity is related to its quantity and function, which are determined by the mutation encoding the defective protein [6]. For example, Class I CFTR mutations result from nonsense, frameshift or splicing mutations in which no functional protein is created. The most common CFTR mutation is F508del, a Class II mutation, which results in protein misfolding, such that it is degraded before it reaches the cell surface. About 85% of the CF patient population carries at least one copy of F508del [7]. CFTR Class III mutations are characterized by defective chloride channel regulation and gating. Mutation Classes I through III are more commonly associated with more severe lung dysfunction and pancreatic insufficiency (PI). Class IV and V mutations are related to defects in channel conductance and stability, respectively; these result in proteins that have residual function and are usually associated with milder disease severity compared to Classes I, II, and III [8]. Absent or dysfunctional CFTR protein in the airways results in the accumulation of thickened secretions that are unable to be cleared by cilia, which leads to chronic airway infections, inflammation, and bronchiectasis. People with CF (PwCF) are known to also suffer from multiple comorbidities beyond respiratory illnesses, including pancreatitis, exocrine and endocrine PI, CF-related diabetes, biliary dysfunction, cirrhosis, intestinal obstruction, malabsorption, nutritional insufficiency, growth delay, nasal polyps, recurrent sinusitis, infertility, anxiety, depression, and many other pathologies [9]. Currently, more than 100,000 people worldwide suffer from the disease and about 1000 individuals are diagnosed each year [10]. 

**Figure 1:**
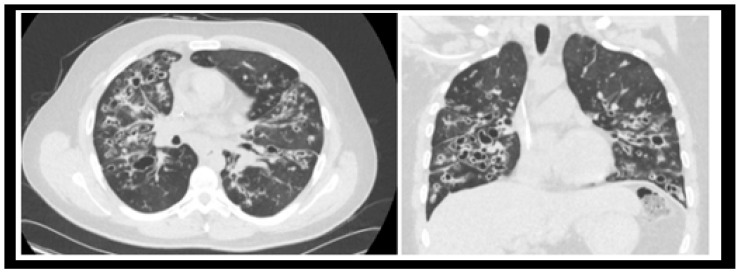
CT radiograph of a patient with advanced CF lung disease demonstrating extensive bronchiectasis and mucoid impaction. Patient was heterozygous for F508del with a forced expiratory volume in 1 s (FEV1) of 0.74 L/22% predicted, history of several pulmonary exacerbations per year, chronic hypoxemia and hypercapnia, and severe pulmonary hypertension. Highly effective modulator therapy, elexacaftor/tezacaftor/ivacaftor, had not yet been approved at the time when the patient required a bilateral lung transplantation.

Although the management of all conditions associated with CF is essential for the complete care of these patients, the pulmonary manifestations of CF have been estimated to account for greater than 90% of the morbidity and mortality of the disease [11]. In the respiratory epithelium, dysfunctional chloride channels lead to the formation of abnormal, viscous fluid on the surface of cells that is insufficiently mobilized by cilia. This leads to a vicious cycle of airway obstruction, infection, and inflammation that progresses to fibrosis resulting in bronchiectasis and ultimately, end-stage lung disease that can only be cured by lung transplantation [12]. Advancements in CF respiratory management include mucolytics, airway hydrators, chest physiotherapy devices, inhalational antimicrobials, and highly effective modulator therapy (HEMT) such as elexacaftor/tezacaftor/ivacaftor (ETI) that correct and improve the underlying protein defect [13]. Undeniably, this led to increased longevity for CF patients. The median life expectancy for CF patients born today now exceeds 50 years of age, whereas it was predicted to be less than 30 in the early 1990s [14]. However, there remains approximately 10% of the United States CF population that is still ineligible for HEMT and continues to have progressive disease [7]. Additionally, people with advanced CF lung disease despite HEMT may still have clinical features associated with worse mortality, warranting lung transplantation evaluation (see Figure 1). Thus, while advancements in CF therapeutics paved the way for an improved life expectancy, there is still no cure for CF, and lung transplantation remains a critical treatment modality.

## 2. Pre-Transplant

### 2.1. Lung Transplantation Referral Criteria in CF

Since the very first lung transplantation procedure was performed in 1963 [15], its implementation has grown dramatically, such that about 4500 lung transplantations are now performed annually across the world [16]. As per guidelines published by the International Society of Heart and Lung Transplantation (ISHLT) [17], ideal lung transplantation recipients have significant lung disease that progressed despite maximal therapy, causes symptoms that limit activities of daily living, and poses a high risk of death over the next two years in the absence of transplantation. However, these patients should not have other significant medical problems and are expected to have at least 80% survival for greater than five years after transplantation. Specifically, although exact contraindications may vary based upon the specializations of a particular transplantation center, individuals who are in shock, require life support, have a detectable HIV viral load, are greater than 70 years old, have limited functional status, are severely overweight (BMI greater than 35) or underweight (BMI less than 16), have had a recent stroke or myocardial infarction, or suffer from stage 3b or greater chronic kidney disease, advanced liver disease, or untreatable hematologic disorders generally are not considered for transplantation. It is strongly recommended to refer eligible patients for evaluation for transplantation early given the extended length of time necessary to complete the workup and to address any concerns before listing a patient. For example, early referral allows patients and caregivers ample time to gain education and to make an informed decision about whether or not to pursue transplantation and to establish a solid relationship with their lung transplant team. For PwCF, it eases transition of care between their CF and transplant care teams. Early referral also allows the transplant team to continuously assess adherence, optimize patients’ overall health and functional status (e.g., weight concerns and deconditioning) and address psychosocial concerns [18]. 

Despite the fatal nature of end-stage lung disease being a shared likeness among many lung diseases, disease-specific indications for lung transplantation are necessary to reflect unique disease characteristics. For example, referral criteria for patients who suffer from chronic obstructive pulmonary disease (COPD) include having a BODE index [19], a composite score measuring body mass index, forced expiratory volume in one second (FEV1), dyspnea, and six-minute walk distance equal to five or six with other features of increased mortality such as an FEV1 that measures less than 25% predicted [17]. A typical referral for those who suffer from interstitial lung disease occurs when they have a forced vital capacity (FVC) that measures less than 80% predicted or a diffusing capacity for carbon monoxide (DLCO) that measures less than 40% predicted, or if they require supplemental oxygen. 

Similar to other diseases, cystic fibrosis carries its own disease-specific indications for referral for lung transplantation (Table 1) long prior to development of terminal disease. According to the Cystic Fibrosis Foundation (CFF) consensus committee [18], specific indications for referral include–an FEV1 that measures less than 50% predicted associated with a greater than 20% relative decline in FEV1 over the previous year, an FEV1 that is less than 40% predicted and is associated with specific markers of shortened survival (greater than two pulmonary exacerbations per year, hemoptysis of greater than 240 mL, pneumothorax, BMI less than 18 despite aggressive attempts to gain weight), or an FEV1 less than 30% predicted even without associated complications. The disease course of CF can be very heterogeneous and, at times, carry an unpredictable nature. In some cases, even preceding lung transplantation referral, the CFF identifies individuals with advanced lung disease, who are at high risk of clinical decline, and, therefore, may benefit from early and robust mitigation of risk factors for disease progression [20]. Specifically, patients who have an FEV1 that measures less than 40% (even without an additional complication), have had a previous intensive care unit admission for respiratory failure, suffer from baseline hypercapnia (PaCO2 greater than 50 on stable blood gas), require oxygen use during the day and at rest, have developed pulmonary hypertension, or cannot walk greater than 400 m would be classified as having advanced lung disease. 

### 2.2. Lung Allocation

The mechanism employed for distribution of lungs for transplantation in the United States changed over the years. Prior to 2005, lungs were distributed according to the duration of time spent on a waitlist. In essence, those people who had been listed for the longest time were those who preferentially received a transplantation. However, pre-transplant mortality was nearly 30% [21]. For that reason, the Lung Allocation Score (LAS) was adopted. It implements a measurement of anticipated survival (in number of days) prior to and following transplantation for each individual. The measurement of survival prior to transplantation is subtracted from that of survival following transplantation to yield a raw score that is normalized on a scale from 0 to 100—the final LAS [22]. Those with the highest LAS are assigned greatest priority for transplantation. In essence, those patients who are in greatest need of transplantation and have the longest anticipated duration of survival with transplantation are the ones who are assigned the greatest priority to receive a transplant, and this system is applied for all indications for lung transplantation. Following implementation of the LAS, the average time spent on waitlists decreased, the total number of transplantations increased, and the average LAS score of patients who underwent transplantation increased [23]. Later on, however, waitlist mortality increased marginally. A suspicion is that, with advancements in treatment for all patients with lung disease, progressively sicker patients have been listed for transplantation, and over-estimation of post-transplant mortality by the LAS may bias against transplantation for these people [24]. 

For PwCF in particular, a recent study showed that median transplant-free survival for patients whose FEV1 measured less than 30% predicted was only 6.6 years [25]. Unfortunately, the median time to transplantation was 8.3 years. Additionally, according to the annual report from the CFF [7], in 2021, 232 PwCF died, but only 54 PwCF underwent lung transplantation. One likely explanation is the insufficient supply of transplantable organs and inadequate referral of patients to transplantation. On average, the lung retrieval rate is lower (15–20%) when compared to other solid organs [26]. Donation after cardiocirculatory death (DCD) accounts for less than 5 percent of the donation pool but recent data showed short- and long-term outcomes after lung transplantation using controlled DCD donation yielded excellent results that were comparable to those of donation after brain death (DBD) [27]. The use of ex vivo lung perfusion (EVLP) as a method to improve organ quality and reverse any injury before transplantation helps to evaluate lungs from uncontrolled DCD and expand the pool of donor lungs available [27]. EVLP use slowly increased overtime, to almost 6% of deceased donor lungs recovered for transplant in the United States since 2015, and there is no evidence of a negative impact on one-year recipient survival [28]. Data are limited regarding the implementation of these tools for CF lung transplantation recipients; however, combined, these approaches are being carried out more frequently to combat an insufficient donor pool. Balancing the acceptable tolerability of donor organ function with transplant outcomes will also help to reduce waitlist mortality. 

As the LAS does not consider factors specific to CF that are associated with increased mortality, such as frequency of CF pulmonary exacerbations, life-threatening hemoptysis, or recurrent pneumothoraces, waitlist mortality for PwCF remained greater than 10% even following implementation of the LAS [29]. Nolley et al. proposed modifications to the LAS to include CF-specific risk factors for mortality. While data are limited, and are not yet validated in an era where use of modulator therapy is more widespread, there is some literature to suggest that use of parameters specific to mortality in CF would increase the predictive value of the LAS for this demographic [30]. 

As of March 2023, a new Composite Allocation Score (CAS) has taken the place of the LAS. Analogous to the LAS, it includes estimates of the mortality prior to, and following, transplantation of a particular person. Unique to the CAS is information about the proximity of the organ donor and recipient to one another (in the interest of maximizing the survival of as many transplantable organs as possible), related to candidates who are of particular consideration for transplantation (patients younger than 18 years of age and those who previously served as organ donors), and concerning candidates who are hard to match with donor organs, such as those whose height or antibody panels are not compatible with most donor organs (in the interest of limiting the time that these people spend on transplant waitlists) [31]. Thus far, based upon data only from simulated patient scenarios, the lung CAS is expected to reduce waitlist deaths and decrease the average time spent on a waitlist while maintaining post-transplant survival [32]. The final new component is of particular relevance to PwCF. Many PwCF likely have suffered from lifelong nutritional deficiencies, intestinal malabsorption, and chronic inflammation, contributing to poor growth rates during childhood and reduced stature as compared to the general population. Although data are yet to come, the lung CAS is expected to lead to a shorter average waitlist time for these individuals. 

## 3. Post-Transplant

In 2021, the CFF released a consensus statement [33] for the care of CF lung transplantation recipients (CFLTRs), as CFLTRs have unique comorbidities that require specialized care. The CFF organized a multidisciplinary committee that formulated 32 recommendations covering topics related to infectious diseases, endocrinology, gastroenterology, pharmacology, mental health, and family planning. A main emphasis was on the close coordination of care between the lung transplantation team, the CF care center, and various sub-specialists with experience in the care of PwCF and lung transplantation recipients. The recommendations of the committee are listed in Table 2. 

## 4. CF Pathogens

A common challenge in lung transplantation in PwCF is the various pathogens seen in the CF respiratory tract [35]—some of which portend worse outcomes (e.g., *Burkholderia cnocepacia*) post transplant. Persistent infections of the allograft lung impose a risk for chronic lung allograft dysfunction (CLAD) [36]. There are two forms of CLAD: bronchiolitis obliterans syndrome (BOS) and restrictive allograft syndrome (RAS). BOS is characterized by an obstructive pulmonary function with radiographic evidence of air trapping on a computed tomography (CT) scan; histopathology demonstrates obliterative bronchiolitis. In RAS, pulmonary function is restrictive, CT demonstrates pleural–parenchymal infiltrates, and pleuroparenchymal fibro-elastosis is seen on histopathology [37]. Certain pathogens such as *Pseudomonas aeruginosa* are significant risk factors for BOS. Thus, isolation of these pathogens from lung transplantation candidates and recipients are heavily considered in their clinical management. Here, we highlight CF pathogens that deserve particular attention during evaluation for, and following, lung transplantation.

### 4.1. Pseudomonas aeruginosa

*Pseudomonas aeruginosa* (PsA) is the most common organism colonizing the airways and sinuses in PwCF. It has been reported in up to 60%, although the prevalence appears to be decreasing since ETI was introduced as HEMT. It is yet to be determined if this is a direct effect of ETI or, more likely, a reflection of poor culture sampling due to diminished sputum production in the majority of PwCF taking ETI [7]. Oftentimes, these bacteria are multi-drug resistant (MDR) given the history of frequent antibiotic treatments that most CF patients receive over the course of their lives for management of pulmonary exacerbations [38]. The PsA strain in CF also tends to be mucoid alginate-producing, which is responsible for the production of a protective biofilm for the bacteria against airway clearance and antibiotic penetrance [39]. The mortality impact of MDR PsA compared to that of more susceptible PsA strains is unclear at this time in CF patients after lung transplantation [40,41]. There are two phases to PsA infection: acute and chronic [42]. Acute pulmonary exacerbations typically present with both pulmonary and systemic symptoms including increased sputum production, increased cough, malaise, and weight loss. Treatment for the acute phase typically includes airway clearance and antibiotics that target lower airway microbes in addition to those that target PsA. In contrast, chronic phase is more indolent and defined by Leed’s criteria as having >50% sputum cultures positive for PsA in the preceding 12 months. Treatments for patients with chronic infections include cycling anti-PsA inhalational antibiotics such as inhaled tobramycin or aztreonam. Intermittent administration of oral and/or intravenous antibiotics is commonly used for disease control [36].

Researchers explored the relationship between post-transplantation bacteria and transplantation outcomes, and preliminary data suggested PsA infection or colonization was likely associated with increased BOS risk [43]. The exact mechanisms remain unknown, but studies in animal models and human lung allografts suggested that PsA colonization in lung allografts may promote activation and migration of lymphocytes into the lung allografts resulting in damages in the lung allograft epithelium [36]. Certain chemokine receptors such as CXCR1 and CXCR2 are integral membrane proteins that bind and respond to cytokines. Studies noted that in PwCF, CXCR1+ lymphocytes were found more frequently, in comparison to patients without CF, suggesting PsA may promote the expansion of CXCR1+ lymphocytes [44]. CXCR1/2 chemokine/ligand axis uses ELR+ chemokines (CXCL1, CXCL5, CXCL7, CXCL8), which are chemotactic for both neutrophils and lymphocytes expressing CXCR1 or CXCR2. Later studies would find that PsA infection, but not colonization, and elevated levels of CXCL1, increased the risk of BOS, with higher levels of CXCL1 furthering the risk [36]. 

### 4.2. Burkholderia cepacia Complex

*Burkholderia cepacia* complex (Bcc) poses a very high risk of reinfection after transplantation and is associated with high mortality rates. Similar to PsA, Bcc infection pre-transplantation persists after transplantation [45]. Bcc comprises over 24 different species. Bcc species most often found in PwCF are *B. cenocepacia*, *B. mutivorans*, *B. vietnamiensis*, *B. dolosa*, *B. cepacia*. While not a part of the Bcc, *B. gladioli* is another *Burkholderia* species often seen in CF [46,47]. Similar to pseudomonas, many strains of Bcc are MDR although Bcc species tend inherently to be resistant to many antibiotic classes. In a cohort of 216 PwCF who underwent lung transplantation, 22 had Bcc pre-transplantation, including 12 with *B cenocepacia*. Nine patients infected with *B. cenocepacia* died post-transplantation, and these deaths were attributed to Bcc sepsis [48]. Colonization with *B. cenocepacia* is commonly a contraindication to lung transplantation in many centers. Bcc carries a heterogeneous virulence; for instance, *B. multivorans* is considered less virulent than *B cenocepacia* and was not associated with a worse survival difference compared to non-infected patients post-transplantation [49]. 

The clinical course of an infection with a Bcc organism can be unpredictable, but the outcome is often related to the pathogenicity of the infecting strain [50]. Bcc typically establishes chronic infection in patients with CF, but can occasionally cause acute necrotizing pneumonia with respiratory failure and sepsis—termed cepacia syndrome. Bcc species tend inherently to be resistant to many antibiotic classes, and as a result, treatment of Bcc is challenging. To date, there are no randomized controlled trials of treatments for respiratory exacerbations in patients with CF chronically infected with Bcc. Thus, there are no optimal antibiotic regimens present [51]. At this time, clinicians must rely on susceptibility data, prior clinical responses and their experience until more data are available. 

### 4.3. Mycobacterium abscessus

*Mycobacterium abscessus* (*M. abscessus*) and mycobacterium avium complex (MAC) are the most common nontuberculous mycobacteria (NTM) found in PwCF [52]. Infection prior to transplantation is associated with an increased risk of recurrence following surgery despite attempts at eradication. *Mycobacterium abscessus* is of particular interest in the field of lung transplantation due to its complication rate post-operatively [53]; for this reason, it may be considered a relative, and sometimes absolute, contraindication in certain transplantation centers [17,18]. Nonetheless, favorable outcomes have been reported in PwCF who are colonized with *Mycobacterium abscessus* and are lung transplantation recipients [54]. Treatment is challenging to tolerate and requires a multidrug treatment regimen. Data on treatment outcomes in PwCF and *Mycobacterium abscessus* lung infections remain limited despite its clinical significance. The American Thoracic Society and the Infectious Disease Society of America recommend a typical treatment schedule divided into two phases: an initial intensive phase and a continuation phase [55]. The intensive phase is composed of an oral macrolide and intravenous amikacin with one or more additional intravenous antibiotics, such as tigecycline, imipenem, or cefoxitin, for a 3–12 week period—depending on severity of infection, response to treatment, and tolerability of the regimen. The continuation phase continues the oral macrolide, and includes inhaled amikacin and two-to-three additional antibiotics such as minocycline, clofazimine, moxifloxacin, or linezolid. The choice of antibiotic should be guided, but not dictated, by drug susceptibility testing. Testing for drug toxicity is essential. Much of this crucial work is carried out through multidisciplinary care and coordination with experts in NTM treatment. 

### 4.4. Aspergillus

*Aspergillus* is a common fungal infection occurring in up to 50% of patients awaiting lung transplantation. *Aspergillus* can demonstrate an array of disease states including asymptomatic colonization, allergic bronchopulmonary aspergillosis, tracheobronchitis, and invasive aspergillosis. Amongst PwCF who have undergone lung transplantation, studies demonstrated that there is a two-to-four-fold higher incidence of *Aspergillus* infections as compared to non-CF patients [56]. Factors that impact airway colonization/infections include direct exposure to the environment, impaired mucociliary clearance, immunosuppression, and donor-transmitted infections. Voriconazole is the drug of choice for invasive aspergillosis, as it was shown to improve outcomes in severely immunosuppressed patients [57]. Other therapies include amphotericin B, caspofungin, and posaconazole, but these treatments are often considered second-line or salvage therapy [58]. Following transplantation, *Aspergillus* is associated with BOS, increases the risk of infections, and impairs oxygenation and ventilation. Due to the risk of BOS, most CF centers will often administer antifungal prophylaxis to their patients after lung transplantation [59]. However, more studies will be required to determine the optimal length of prophylactic therapy balancing the benefit of prophylaxis with the risks of drug toxicities. 

### 4.5. COVID-19

Over the course of the past several years, the pandemic of COVID-19 infection undoubtedly devastated several patient populations across the entire world. Early in the pandemic, initial reports [60] and subsequent data [61] alike suggested that rates of infection among PwCF were not significantly higher than those of the general population. Although altered pulmonary anatomy and physiology might have mechanistically suggested a predisposition to infection, possible protective factors for PwCF include use of airway clearance medications and pre-existent knowledge about infection control practices and social distancing. Nonetheless, COVID-19 infection can predispose PwCF to pulmonary exacerbations and can lead to severe, life-threatening disease, particularly in lung transplant recipients who are immunosuppressed [61].

## 5. Noninfectious Complications

Noninfectious complications unique to CF patients after lung transplantation include endocrine, gastrointestinal, and oncologic pathologies. CF patients at baseline are uniquely susceptible to the development of insulin deficiency, and after transplant due to the use of corticosteroids and immunosuppressive therapies, the prevalence of diabetes mellitus increases [62]. Post-transplantation status and corticosteroid exposure also predispose to accelerated bone loss, pathological fractures, as well as osteonecrosis [63]. Epstein–Barr Virus mismatch, in the context of chronic immunosuppressive therapy, increases the risk for malignancy, particularly post-transplant lymphoproliferative disease (PTLD). Cancer screening is discussed in more detail in the article entitled “Future Comorbidities in an Aging Cystic Fibrosis Population”.

Gastrointestinal complications include gastroesophageal reflux and intestinal dysmotility. These conditions are common in CF, and they may worsen after transplantation [64]. Due to inherent dysmotility, intestinal obstruction syndrome may occur and result in further malnutrition which is an important risk factor for poor outcomes after surgery [65]. Distal intestinal obstruction syndrome (DIOS) occurs in 15.9% of adults with CF and increased risk of gastrointestinal complications such as DIOS is well known after lung transplantation [64]. Differentiating DIOS from other causes of bowel obstruction, such as constipation, in CF may be difficult, but one of the more widely used definitions for DIOS is an acute complete, or incomplete, fecal obstruction in the ileocecum. Constipation, however, is defined as gradual fecal impaction of the total colon [66]. Studies have shown that the incidence of DIOS post lung transplantation is roughly 10% [67]. Factors that are theorized to contribute to this syndrome include pancreatic insufficiency, reduced intestinal water content, slow intestinal transit, and dehydration of the mucus layer due to altered intestinal secretion [68]. Additionally, pre-transplantation abdominal operation and prior meconium ileus were some of the strongest predictors of developing DIOS [67]. Preventive therapy for DIOS includes the implementation of oral laxatives, adequate hydration, and pancreatic enzyme supplementation; however, there is a lack of consensus regarding the best preventive measures [69]. 

## 6. Prognosis

The median survival of CF patients after lung transplant is 8.3 years, whereas it is 5.7 years after lung transplant for all indications [70]. Although lung transplant is the standard of care for CF patients with end-stage lung disease, and although predictors of mortality prior to transplant are well defined, factors that prognosticate mortality following transplant in CF are not as well established. Risk stratifications to determine prospective lung transplant candidates who would benefit most from transplantation can be challenging [71]. A systematic review and meta-analysis evaluated risk factors of age, gender, FEV1, pulmonary hypertension, BMI, CF-related diabetes, and pre-existing infections with Bcc and/or PsA with post-transplant mortality in CF [72]. The single risk factor associated with increased mortality after transplantation was pre-existent Bcc infection. As discussed previously, the species of Bcc have varying virulence, and so, this must be taken into account; *B. cenocepacia* is associated with worse mortality. Thus, it is vital to identify species of microorganisms that are commonly identified within a complex. Surprisingly, factors that are known to be associated with waitlist mortality, such as low FEV1, pulmonary hypertension, low BMI, female gender, and CF-related diabetes, were not associated with post-transplant mortality. Therefore, the use of existing risk stratification tools may actually underestimate the survival benefit of transplantation in CF. The most likely reason that low FEV1 and pulmonary hypertension do not affect the mortality after transplant is that the surgery leads to the improvement of these two factors. 

For some time, low BMI was believed to be associated with poorer outcomes in CF lung transplantation recipients. The theory was that worsening CF with advanced lung disease is correlated with reduced nutrition as patients will have dyspnea, anorexia, and increased basal energy expenditure from chronic respiratory infections. Early data showed that underweight status prior to lung transplantation was associated with mortality after transplantation [73]. Therefore, low BMI was frequently one of the absolute contraindications to lung transplantation [74]. Challenging this idea, recent data from the United Network for Organ Sharing (UNOS) dataset revealed that the median survival in patients with CF and BMI < 17 kg/m^2^ was 7.0 years, which was comparable to subjects with other commonly transplanted diagnoses [75]. A likely reason for this disparity between theoretical risk and clinical outcomes data is that BMI does not differentiate between fat mass and fat-free body mass. In comparison, sarcopenia may be a more useful prognostic indicator. One study demonstrated that decreased muscle mass index, as measured by CT scan at the time of lung transplantation, was associated with decreased survival and increased hospital length of stay after transplantation [76]. Another study similarly showed that low muscle cross-sectional area, as measured by CT scan, was associated with increased duration of hospitalization after transplantation [77]. Although pre-transplantation FEV1 has not been associated with post-transplant mortality, as described previously, low FEV1 is a marker for severe disease. Thus, as recent data demonstrated that sarcopenia prior to transplantation is a predictor of poor outcomes subsequent to transplantation, it is understandable also that skeletal muscle mass index prior to transplantation is associated directly with FEV1 [78]. Another explanation for the lack of correlation between pre-transplant BMI and post-transplant outcome may be that nutritional status is reversible. Underweight patients with CF were shown to have higher risk of death without lung transplantation, highlighting the urgency for transplantation [75]. These new data challenge pre-existing beliefs and highlight that patients with CF and low BMI may be unjustifiably excluded from lung transplantation.

Although transplantation in patients with CF, as compared with transplantation in the context of other end-stage lung diseases, is associated with relatively favorable outcomes, CF patients tend to be quite young at the time of transplantation. For that reason, eventually, some CF patients will develop CLAD, but they may continue to have relatively limited medical comorbidities, so they may be eligible for an additional lung transplant. CLAD occurs in about 50% of recipients 5 years after lung transplantation [79]. Unfortunately, the survival rates of re-transplantation remain significantly worse, compared to those of primary transplantation despite advances in immunosuppression and post-operative management [80]. The risk factors associated with developing CLAD remain unclear. What has been shown is that patients who require repeat transplantation often experience a decline in clinical status across multiple organ systems [80]. The more notable organs include the renal system, with higher baseline creatinine, and the pulmonary system with increased oxygen requirements or lower FEV1 [80]. Due to these organ failures the patients are often bridged with dialysis for their kidneys or with mechanical ventilation and/or extracorporeal membrane oxygenation (ECMO) for their pulmonary/circulatory failure. It is important to note, however, that, in general, patients who previously have undergone lung transplantation are at about a five-fold increased risk for all-cause mortality, as compared to the general population [81]. Additionally, these forms of external organ support were noted as predictors of worsening cardiopulmonary status and graft failure three years after transplantation [82] and, thus, are likely only further to exacerbate this known mortality risk. It is also important to note that, as compared to patients undergoing a primary lung transplantation, those who ultimately require a re-transplantation require a greater duration of mechanical ventilation and, on average, have a longer post-transplantation ICU stay [83]. Ultimately, these individuals have worse pulmonary function than those undergoing a first transplantation [83], and some single-center studies even noted greater mortality [84,85]. Therefore, earlier identification of patients who are at higher risk of needing another transplantation is imperative, as it may allow for earlier intervention and, thereby, alleviate the need to bridge these patients to transplantation with mechanical ventilation or ECMO. It also may allow for the optimization of patients such that they are in better condition prior to the re-transplantation surgery. In particular, preliminary data regarding the use of inhaled cyclosporine are promising. An initial study of 58 patients published in 2006 demonstrated that inhaled cyclosporine administered prophylactically to lung transplantation recipients did not decrease the incidence of acute rejection, but it did increase the duration of both survival and chronic, rejection-free survival [86]. A subsequent phase II study regarding the use of inhaled cyclosporine in patients who developed BOS demonstrated a decreased incidence of progression of BOS grade in the treatment arm [87]. Data from phase III randomized controlled trials of inhaled cyclosporine use in patients who suffer from BOS, both in the context of single lung transplantation [88] and double lung transplantation [89], are pending. Given these promising data that demonstrate potential for intervention to mitigate the need for re-transplantation, it is of utmost urgency to determine risk factors associated with the development, and worsening, of CLAD. The exact predisposing features associated with CLAD remain elusive, but some studies associated thymoglobuin use, number of infections treated, and certain infections with increased incidence of CLAD [37]. However, additional studies are needed to identify the specific risk factors associated with the development of CLAD. 

## 7. The Changing Landscape of Lung Transplantation in CF

The average age of patients with CF undergoing lung transplantation has been rising—arguably as a result of implementation of modulator therapy and improved comprehensive care of these patients. The percentage of pediatric patients among those who received a lung transplant for CF decreased from 12.5% between 2005 and 2009 to 9.6% from 2010 to 2014, and the percentage of patients aged greater than 40 years increased 14.2% to 17.2% over the same time period [90]. According to pooled data from the UNOS from 1992 to 2016, of all PwCF who underwent lung transplantation, those patients who were older than 30 years had a significantly greater median survival as compared to those whose ages were between 18 and 29 years old [90]. Secondary analysis demonstrated that the younger group had higher allograft failure as well as higher incidence of malignancy. With the increasing age of CF lung transplant recipients, one could hypothesize that this could translate to improvement in survival post-transplantation. However, older aged patients may also carry additional age-related comorbidities. Further studies are needed to elucidate the underlying mechanisms of age-related disparity in survival, but there are a few hypotheses [91]. The older recipients may have had phenotypically milder CF; thus, they did not require a transplantation until they reached an older age. However, this study was performed over a relatively long period of time, over which the average age of lung transplantation recipients did increase dramatically–possibly due to external factors such as ongoing improvements in standardized care, thereby delaying the need for transplantation. Behavioral maturation, associated with better medication adherence and psychosocial support, is another possibility for the observed difference in survival between the age groups. Furthermore, younger-aged patients typically had public insurance, in contrast to private insurance most commonly seen in older patients, which may have contributed to their level of access to care. Another observation is that cytomegalovirus (CMV) and Epstein–Barr virus (EBV) recipient sero-negative status was more likely to occur in younger patients, resulting in a higher chance for CMV and EBV mismatch if the donor was sero-positive, thereby increasing the risk of the development of post-transplant lymphoproliferative disorders. 

## 8. HEMT Impact on Transplantation Landscape

The first FDA-approved modulator was ivacaftor, an oral agent which targeted a point mutation, G551D, in the CFTR. Mutations in G551D result in abnormal CFTR proteins that have an impaired ability to open. Ivacaftor allows the channel opening probability to increase and, therefore, is classified as a potentiator. Early studies conducted by Accurso and colleagues [92], which evaluated the safety of this medication, found in their secondary endpoints that there were also changes in predicted FEV1 and median sweat chloride levels. Later CFTR modulator studies would also measure these changes. Although there have been studies that demonstrated no correlation between reductions in FEV1 and mean sweat chloride level, with respect to an individual patient, in the context of ivacaftor use, when these studies were pooled together with respect to a population, sweat chloride level changes did appear to correlate with lung function changes [93].

Since the approval of ivacaftor, there have been other potentiators as well as correctors that were approved for CF. Further clinical trials demonstrated that a combination of potentiators and correctors are more efficacious than monotherapy alone [93]. The newest FDA-approved combination therapy is elexacaftor/tezacaftor/ivacaftor (ETI), the first triple combination therapy, for patients who are 12 years and older that have CF with at least one F508del mutation in the CFTR protein. 

The development of HEMT such as ETI revolutionized the management not only of CF patients over the course of their lifespan but also of those with advanced lung disease. It is important to recognize that although CF patients no longer suffer from CF lung disease after transplantation, these patients continue to suffer from systemic complications of CF. Previously, it was theorized that patients who had undergone lung transplant would not benefit from HEMT because the primary end point for modulator therapy trials was commonly the change in FEV1% predicted. However, recent studies demonstrated that the use of HEMT, even after transplantation, helps to manage important non-pulmonary manifestations of CF, including nutritional and gastrointestinal outcomes, sinus disease, and persistent pseudomonal infections [94]. Other notable considerations in using ETI following transplantation include observational data demonstrating no impact on graft dysfunction [95] or on immunosuppression drug regimen/dosing [96]. As a result, the use of HEMT post-lung transplant may be of potential benefit with acceptable safety profiles. Nonetheless, clinicians must be vigilant and monitor for side effects and toxicities of HEMT including drug–drug [96] interactions, even though relative safety in combination with immunotherapy was observed. More research is needed before further recommendations can be made regarding the use of HEMT post lung transplant. 

## 9. Conclusions

While CF is a rare disease, it has the potential to cause significant morbidity and mortality to those with advanced pathology, and the greatest suffering in CF is associated with pulmonary disease. The only cure for end-stage lung disease is pulmonary transplantation. Despite the hurdles specific to coordination of lung transplantation for CF patients, its success in this demographic is evident. The pathology central to CF was only first described in a case series in 1938 [97], and its gene was only identified in 1989 [98]. Nonetheless, lung transplantation for CF has been performed since 1983 [99], and CF now accounts for about 17% of pre-transplant diagnoses [70]. The median survival of CF patients after lung transplant is 8.3 years, whereas it is 5.7 years after lung transplant for all indications [70]. 

Although HEMT continues to demonstrate promise in mitigating the symptoms of, and progression to, severe disease, it cannot be used to treat all illness-causing mutations, and some patients suffer from disease that already advanced greatly. Thus, it is likely that lung transplantation will remain a cornerstone of treatment of terminal pulmonary disease in CF for years to come. 

However, there are still a lot of advances to be made to ensure that as many eligible patients as possible survive to the time of transplantation—particularly via modification of the lung allocation scoring system for CF patients. With ongoing advancements in care for CF patients, both before and after transplant, particularly due to the implementation of HEMT, pre- and post-transplant longevity of CF patients is likely to improve. For that reason, future research may focus on re-defining indications or contraindications for transplantation in this patient population. For instance, disease complications, such as suboptimal BMI, or infections with highly virulent organisms, which may previously have been deemed contraindications to transplantation, may represent stronger indications for transplantation in the future. Additionally, investigation might focus on longevity post transplantation, CLAD, and indications for re-transplantation, as the population of long-term survivors after transplantation grows. 

## Figures and Tables

**Table 1:** Indications and Contraindications for Lung Transplantation and CF-Specific Factors [17,18,20].

General Indications	-Advanced lung disease that is refractory to medical therapy -Greater than 50% mortality from lung disease without transplantation over the next two years -Greater than 80% five-year survival post-transplantation
Absolute Contraindications	-Clinical shock, disseminated infection, or HIV infection with a detectable viral load -Malignancy with high risk of recurrence or cancer-related death -Lack of interest in transplantation, evidence of persistent non-adherence to treatment -Non-pulmonary organ dysfunction -GFR less than 40 -Stroke or acute coronary syndrome within the past thirty days -Acute liver failure or cirrhosis with synthetic dysfunction -Hematologic disorders not amenable to treatment -Active substance use or dependence
Relative Contraindications	-Age greater than 70 years -Severe coronary artery disease or cerebrovascular disease -BMI greater than 35 or less than 16 kg/m^2^ -Severe esophageal dysmotility, chest wall deformity, or previous thoracic surgery expected to cause difficulty with post-transplant healing -Infection or colonization with highly resistant or virulent organism -Detectable hepatitis B or C viral load -Limited functional status or neurocognitive condition that may interfere with adherence to regimen after transplantation
CF-Specific Referral Criteria	-FEV1 < 50% predicted with a greater than 20% relative decline in FEV1 within one year -FEV1 < 40% predicted accompanied by: -More than two exacerbations per year requiring antibiotics -Massive hemoptysis of more than 240 mL that necessitates ICU admission or bronchial artery embolization -BMI less than 18 -FeV1 < 30% predicted -Six-minute walk test result < 400 m -Advanced CF lung disease: -Hypoxia (PaO2 < 55 mmHg) at rest or with exertion -Hypercapnia (PaCO2 > 50 mmHg) -Previous exacerbation requiring positive pressure ventilation -Pulmonary artery systolic pressure > 50 mmHg

**Table 2:** CFF recommendations for the care of PwCF after lung transplantation.

Category	Recommendation	% Vote
General Care	CF Lung Transplant Recipients should follow up with a multidisciplinary CF care team within 6–12 months of transplant to resume extra-pulmonary CF care. Communication between the transplant and CF care teams is essential for coordination of care	100%
General Care	CF and Transplant programs should operationalize infection prevention and control policies across all services as indicated by the CF Foundation’s Infection Prevention and Control Guidelines	95%
Infectious disease	Non–invasive CF-specific bacterial, fungal, and AFB respiratory cultures should be obtained by the transplant or CF center every 3 months in actively waitlisted transplant candidates, and clinicians should review prior pathogen history to guide the peri-operative antibiotic regimen	100%
Infectious disease	An intraoperative CF bacterial, fungal and AFB culture of the native lung should be obtained at the time of lung transplantation	100%
Infectious disease	In CF Lung Transplant Recipients with multidrug resistant pathogens, susceptibility-driven antimicrobials should be administered when the recipient has a susceptible antibiotic choice with acceptable toxicity. In the absence of a susceptibility-driven perioperative choice, previously effective regimens should be considered	100%
Infectious disease	For CF Lung Transplant Recipients, there are insufficient evidence to recommend for or against routine intraoperative pleural and tracheal irrigation with antimicrobial agents to decrease infections after transplant	100%
Infectious disease	Perioperative and/or early posttransplant inhaled antibiotics for bacterial pathogens isolated prior to transplant should be considered as a complement to systemic antimicrobials in CF Lung Transplant Recipients	100%
Infectious disease	There is insufficient evidence to recommend for or against the use of inhaled antibiotics for prevention of recolonization or chronic lung allograft dysfunction (CLAD)	100%
Infectious disease	There is insufficient evidence to recommend for or against the routine collection of sputum for bacterial, fungal or AFB cultures in asymptomatic CF Lung Transplant Recipients	95%
Sinus Disease	In individuals with CF and asymptomatic chronic rhinosinusitis (CRS), the CF Foundation recommends against pre-transplant prophylactic sinus surgery for the prevention of lung graft colonization.	100%
Sinus Disease	CF Lung Transplant Recipients should be screened for symptoms of CRS at least annually	100%
Sinus Disease	CF Lung Transplant Recipients with moderate or severe symptomatic CRS should be seen in consultation with an otolaryngologist experienced in CF for consideration of optimal topical therapies and endoscopic sinus surgery	100%
Nutrition and GI Complications	CF Lung Transplant Recipients should receive ongoing consultation with a dietitian with CF expertise in order to obtain individualized nutritional therapy to achieve an established BMI or weight-for-length goal	100%
Nutrition and GI Complications	In CF Lung Transplant Recipients, vitamin D supplementation should be continued, but a combination of vitamin A,D,E,K supplements should be discontinued after lung transplantation; fat soluble vitamin levels should be measured by 3 months after transplant, and levels should be repleted and followed individually as needed	100%
Nutrition and GI Complications	Symptoms should be assessed daily in hospitalized patients, particularly within the immediate post-operative period and with any opiate medication administration, for early signs of obstipation and obstruction that might herald emergence of distal intestinal obstruction syndrome (DIOS)	100%
Nutrition and GI Complications	In CF Lung Transplant Recipients who develop DIOS, early enteral lavage should be considered. Refractory DIOS should be managed in coordination with experts in CF gastrointestinal complications to reduce risk for prolonged obstruction and potential need for operative management	100%
Nutrition and GI Complications	For CF Lung Transplant Recipients who experience new or worsening symptoms of gastrointestinal dysmotility, a gastroenterologist and a dietitian with CF expertise should be consulted to guide the approach to symptom control and potential interventions	100%
Nutrition and GI Complications	CF Lung Transplant Recipients should undergo liver enzyme monitoring for CF Liver Disease (CFLD) at least annually, and when levels are elevated, patients should receive non-invasive imaging techniques for initial evaluation	
Diabetes and Bone Health	CF Lung Transplant Recipients who do not have Cystic Fibrosis-Related Diabetes (CFRD) should be screened with an oral glucose tolerance test (OGTT) at 3–6 months after transplant and then annually–following the recommended screening guidelines for CFRD	95%
Diabetes and Bone Health	CF Lung Transplant Recipients who have CFRD should be treated with insulin and should undergo intensive self-blood glucose monitoring (SBGM) and individualized close clinical follow-up in addition to lifestyle modifications. Furthermore, an endocrinologist with expertise in CF and transplant associated DM should be consulted when possible	
Diabetes and Bone Health	For CF Lung Transplant Recipients, bone density should be assessed with dual energy X-ray absorptiometry (DEXA) at 6–12 months after transplant	100%
Mental Health and Family Planning	CF Lung Transplant Recipients should have mental health screening and consultation for depression, anxiety, and post-traumatic stress disorder (PTSD) within *6* months of transplant and then should resume annual screening per the International Committee on Mental Health (ICMH) Depression and Anxiety Guidelines	100%
Mental Health and Family Planning	Caregivers of CF Lung Transplant Recipients should be screened for depression, anxiety, and PTSD within 6 months of transplant and should be referred for further assessment if necessary	90%
Mental Health and Family Planning	Females with CF who are post-lung transplant and are considering pregnancy should assess carefully their individual risks through shared decision making with maternal fetal medicine and transplant providers	100%
Mental Health and Family Planning	Females with CF who are post-lung transplant should avoid pregnancy for at least the first 2 years after transplantation because of the increased risk of acute rejection, accelerated chronic rejection, and death	100%
Pharmacology and Therapeutics	There is insufficient evidence to recommend for or against the use of Cystic Fibrosis Transmembrane Conductance Regulator (CFTR) modulators for CF Lung Transplant Recipients	100%
Pharmacology and Therapeutics	There is insufficient evidence to recommend for or against the use of induction immunosuppression for CF Lung Transplant Recipients	100%
Pharmacology and Therapeutics	CF Lung Transplant Recipients should have close monitoring of calcineurin inhibitor drug levels because of altered pharmacokinetics	100%
Pharmacology and Therapeutics	Reduced renal function is common in CF Lung Transplant Recipients, and serum creatinine is often a poor surrogate for renal function. Therefore, medications should be dosed carefully according to the estimated glomerular filtration rate (GFR) of the patient, and when available, therapeutic drug monitoring should be implemented	100%
Pharmacology and Therapeutics	There is insufficient evidence to recommend for or against the routine use of airway clearance, dornase alfa, or hypertonic saline among CF Lung Transplant Recipients	100%

Note. Reprinted from Table 1 in [34]. Creative Commons user license link: https://creativecommons.org/licenses/by/4.0/ accessed on 10 April 2023.

## Data Availability

Not applicable.

## References

[1] Bobadilla J.L., Macek M., Fine J.P., Farrell P.M. (2002). Cystic fibrosis: A worldwide analysis of CFTR mutations—correlation with incidence data and application to screening. Hum. Mutat..

[2] Saint-Criq V., Gray M.A. (2017). Role of CFTR in epithelial physiology. Cell. Mol. Life Sci..

[3] Yoshimura K., Nakamura H., Trapnell B.C., Chu C.-S., Dakemans W., Pavirani A., Lecocq J.-P., Crystal R.G. (1991). Expression of the cystic fibrosis transmembrane conductance regulator gene in cells of non-epithelial origin. Nucleic Acids Res..

[4] Xue R., Gu H., Qiu Y., Guo Y., Korteweg C., Huang J., Gu J. (2016). Expression of Cystic Fibrosis Transmembrane Conductance Regulator in Ganglia of Human Gastrointestinal Tract. Sci. Rep..

[5] Collawn J.F., Matalon S. (2014). CFTR and lung homeostasis. Am. J. Physiol. Cell. Mol. Physiol..

[6] De Boeck K., Amaral M.D. (2016). Progress in therapies for cystic fibrosis. Lancet Respir. Med..

[7] Cystic Fibrosis Foundation Patient Registry 2021 Annual Data Report. Bethesda, Maryland. https://www.cff.org/sites/default/files/2021-11/Patient-Registry-Annual-Data-Report.pdf.

[8] Wang J., Cohen R.I. (2020). Cystic Fibrosis Therapy: From Chest Physiotherapy to Agents Targeting Specific Mutations. Eur. J. Respir. Med..

[9] Hamosh A., FitzSimmons S.C., Macek M., Knowles M.R., Rosenstein B.J., Cutting G.R. (1998). Comparison of the clinical manifestations of cystic fibrosis in black and white patients. J. Pediatr..

[10] About Cystic Fibrosis|Cystic Fibrosis Foundation. https://www.cff.org/intro-cf/about-cystic-fibrosis.

[11] Belkin R.A., Henig N.R., Singer L.G., Chaparro C., Rubenstein R.C., Xie S.X., Yee J.Y., Kotloff R.M., Lipson D.A., Bunin G.R. (2006). Risk Factors for Death of Patients with Cystic Fibrosis Awaiting Lung Transplantation. Am. J. Respir. Crit. Care Med..

[12] Bergeron C., Cantin A.M. (2019). Cystic Fibrosis: Pathophysiology of Lung Disease. Semin. Respir. Crit. Care Med..

[13] Castellani C., Duff A.J., Bell S.C., Heijerman H.G., Munck A., Ratjen F., Sermet-Gaudelus I., Southern K.W., Barben J., Flume P.A. (2018). ECFS best practice guidelines: The 2018 revision. J. Cyst. Fibros..

[14] Understanding Changes in Life Expectancy|Cystic Fibrosis Foundation. https://www.cff.org/managing-cf/understanding-changes-life-expectancy.

[15] Griscom N.T. (1963). Lung transplantation. JAMA.

[16] Chambers D.C., Perch M., Zuckermann A., Cherikh W.S., Harhay M.O., Hayes D., Hsich E., Khush K.K., Potena L., Sadavarte A. (2021). The International Thoracic Organ Transplant Registry of the International Society for Heart and Lung Transplantation: Thirty-eighth adult lung transplantation report—2021; Focus on recipient characteristics. J. Heart Lung Transplant..

[17] Leard L.E., Holm A.M., Valapour M., Glanville A.R., Attawar S., Aversa M., Campos S.V., Christon L.M., Cypel M., Dellgren G. (2021). Consensus document for the selection of lung transplant candidates: An update from the International Society for Heart and Lung Transplantation. J. Heart Lung Transplant..

[18] Ramos K.J., Smith P.J., McKone E.F., Pilewski J.M., Lucy A., Hempstead S.E., Tallarico E., Faro A., Rosenbluth D.B., Gray A.L. (2019). Lung transplant referral for individuals with cystic fibrosis: Cystic Fibrosis Foundation consensus guidelines. J. Cyst. Fibros..

[19] Celli B.R., Cote C.G., Marin J.M., Casanova C., Montes de Oca M., Mendez R.A., Plata V.P., Cabral H.J. (2004). The Body-Mass Index, Airflow Obstruction, Dyspnea, and Exercise Capacity Index in Chronic Obstructive Pulmonary Disease. N. Engl. J. Med..

[20] Kapnadak S.G., Dimango E., Hadjiliadis D., Hempstead S.E., Tallarico E., Pilewski J.M., Faro A., Albright J., Benden C., Blair S. (2020). Cystic Fibrosis Foundation consensus guidelines for the care of individuals with advanced cystic fibrosis lung disease. J. Cyst. Fibros..

[21] De Meester J., Smits J.M., Persijn G.G., Haverich A. (2001). Listing for lung transplantation: Life expectancy and transplant effect, stratified by type of end-stage lung disease, the Eurotransplant experience. J. Heart Lung Transplant..

[22] Davis S.Q., Garrity E.R. (2007). Organ Allocation in Lung Transplant. Chest.

[23] Egan T.M., Edwards L.B. (2016). Effect of the lung allocation score on lung transplantation in the United States. J. Heart Lung Transplant..

[24] Brahmbhatt J.M., Wai T.H., Goss C.H., Lease E.D., Merlo C.A., Kapnadak S.G., Ramos K.J. (2022). The lung allocation score and other available models lack predictive accuracy for post-lung transplant survival. J. Heart Lung Transplant..

[25] Ramos K.J., Quon B.S., Heltshe S.L., Mayer-Hamblett N., Lease E.D., Aitken M.L., Weiss N.S., Goss C.H. (2017). Heterogeneity in Survival in Adult Patients with Cystic Fibrosis With FEV1 < 30% of Predicted in the United States. Chest.

[26] Keshavamurthy S., Rodgers-Fischl P. (2021). Donation after circulatory death (DCD)—Lung procurement. Indian J. Thorac. Cardiovasc. Surg..

[27] Inci I. (2017). Donors after cardiocirculatory death and lung transplantation. J. Thorac. Dis..

[28] Lehman R., Uccellini K., Lease E., Daly R., Chan K. (2019). Increasing Use of EVLP in the United States: Data from the OPTN/UNOS. J. Heart Lung Transplant..

[29] Nolley E.P., Pilewski J.M. (2019). Call for Changes in Lung Allocation to Reduce Transplant Wait-List Mortality for Cystic Fibrosis. Am. J. Respir. Crit. Care Med..

[30] Lehr C.J., Skeans M., Dasenbrook E., Fink A., Fernandez G., Faro A., Valapour M. (2019). Effect of Including Important Clinical Variables on Accuracy of the Lung Allocation Score for Cystic Fibrosis and Chronic Obstructive Pulmonary Disease. Am. J. Respir. Crit. Care Med..

[31] Lung Allocation Based on the Composite Allocation Score (CAS): Questions and Answers for Patients and Caregivers—OPTN. https://optn.transplant.hrsa.gov/patients/by-organ/lung/lung-allocation-based-on-the-composite-allocation-score-cas-questions-and-answers-for-patients-and-caregivers/.

[32] Valapour M., Lehr C.J., Wey A., Skeans M.A., Miller J., Lease E.D. (2022). Expected effect of the lung Composite Allocation Score system on US lung transplantation. Am. J. Transplant..

[33] McKone E., Ramos K.J., Chaparro C., Blatter J., Hachem R., Anstead M., Vlahos F., Thaxton A., Hempstead S., Daniels T. (2023). Position paper: Models of post-transplant care for individuals with cystic fibrosis. J. Cyst. Fibros..

[34] Shah P., Lowery E., Chaparro C., Visner G., Hempstead S.E., Abraham J., Bhakta Z., Carroll M., Christon L., Danziger-Isakov L. (2021). Cystic fibrosis foundation consensus statements for the care of cystic fibrosis lung transplant recipients. J. Heart Lung Transplant..

[35] Kotloff R.M., Zuckerman J.B. (1996). Lung Transplantation for Cystic Fibrosis. Chest.

[36] Gregson A.L. (2016). Infectious Triggers of Chronic Lung Allograft Dysfunction. Curr. Infect. Dis. Rep..

[37] Verleden S.E., Vos R., Vanaudenaerde B.M., Verleden G.M. (2017). Chronic lung allograft dysfunction phenotypes and treatment. J. Thorac. Dis..

[38] Soetanto V., Grewal U.S., Mehta A.C., Shah P., Varma M., Garg D., Majumdar T., Dangayach N.S., Grewal H.S. (2022). Early postoperative complications in lung transplant recipients. Indian J. Thorac. Cardiovasc. Surg..

[39] Govan J.R., Deretic V. (1996). Microbial pathogenesis in cystic fibrosis: Mucoid Pseudomonas aeruginosa and *Burkholderia cepacia*. Microbiol. Rev..

[40] Dales L., Ferris W., Vandemheen K., Aaron S.D. (2009). Combination antibiotic susceptibility of biofilm-grown *Burkholderia cepacia* and Pseudomonas aeruginosa isolated from patients with pulmonary exacerbations of cystic fibrosis. Eur. J. Clin. Microbiol. Infect. Dis..

[41] Hadjiliadis D., Steele M.P., Chaparro C., Singer L.G., Waddell T.K., Hutcheon M.A., Davis R.D., Tullis D.E., Palmer S.M., Keshavjee S. (2007). Survival of Lung Transplant Patients with Cystic Fibrosis Harboring Panresistant Bacteria Other Than *Burkholderia cepacia*, Compared with Patients Harboring Sensitive Bacteria. J. Heart Lung Transplant..

[42] Sousa A.M., Pereira M.O. (2014). Pseudomonas aeruginosa Diversification during Infection Development in Cystic Fibrosis Lungs—A Review. Pathogens.

[43] Gottlieb J., Mattner F., Weissbrodt H., Dierich M., Fuehner T., Strueber M., Simon A., Welte T. (2009). Impact of graft colonization with gram-negative bacteria after lung transplantation on the development of bronchiolitis obliterans syndrome in recipients with cystic fibrosis. Respir. Med..

[44] Takata H., Tomiyama H., Fujiwara M., Kobayashi N., Takiguchi M. (2004). Cutting Edge: Expression of Chemokine Receptor CXCR1 on Human Effector CD8+ T Cells. J. Immunol..

[45] Dobbin C., Maley M., Harkness J., Benn R., Malouf M., Glanville A., Bye P. (2004). The impact of pan-resistant bacterial pathogens on survival after lung transplantation in cystic fibrosis: Results from a single large referral centre. J. Hosp. Infect..

[46] Scoffone V.C., Chiarelli L.R., Trespidi G., Mentasti M., Riccardi G., Buroni S. (2017). Burkholderia cenocepacia Infections in Cystic Fibrosis Patients: Drug Resistance and Therapeutic Approaches. Front. Microbiol..

[47] Somayaji R., Yau Y.C.W., Tullis E., LiPuma J.J., Ratjen F., Waters V. (2020). Clinical Outcomes Associated with *Burkholderia cepacia* Complex Infection in Patients with Cystic Fibrosis. Ann. Am. Thorac. Soc..

[48] De Soyza A., Meachery G., Hester K.L., Nicholson A., Parry G., Tocewicz K., Pillay T., Clark S., Lordan J.L., Schueler S. (2010). Lung transplantation for patients with cystic fibrosis and *Burkholderia cepacia* complex infection: A single-center experience. J. Heart Lung Transplant..

[49] Chaparro C., Maurer J., Gutierrez C., Krajden M., Chan C., Winton T., Keshavjee S., Scavuzzo M., Tullis E., Hutcheon M. (2001). Infection with *Burkholderia cepacia* in Cystic Fibrosis: Outcome following lung transplantation. Am. J. Respir. Crit. Care Med..

[50] France M.W., Dodd M.E., Govan J.R., Doherty C.J., Webb A., Jones A.M. (2008). The changing epidemiology of Burkholderia species infection at an adult cystic fibrosis centre. J. Cyst. Fibros..

[51] Lord R., Jones A.M., Horsley A. (2020). Antibiotic treatment for *Burkholderia cepacia* complex in people with cystic fibrosis experiencing a pulmonary exacerbation. Cochrane Database Syst. Rev..

[52] Nontuberculous Mycobacteria (NTM)|Cystic Fibrosis Foundation. https://www.cff.org/managing-cf/nontuberculous-mycobacteria-ntm.

[53] Chalermskulrat W. (2006). Non-tuberculous mycobacteria in end stage cystic fibrosis: Implications for lung transplantation. Thorax.

[54] Raats D., Lorent N., Saegeman V., Vos R., van Ingen J., Verleden G., Van Raemdonck D., Dupont L. (2019). Successful lung transplantation for chronic *Mycobacterium abscessus* infection in advanced cystic fibrosis, a case series. Transpl. Infect. Dis..

[55] Daley C.L., Iaccarino J.M., Lange C., Cambau E., Wallace R.J., Andrejak C., Böttger E.C., Brozek J., Griffith D.E., Guglielmetti L. (2020). Treatment of Nontuberculous Mycobacterial Pulmonary Disease: An Official ATS/ERS/ESCMID/IDSA Clinical Practice Guideline: Executive Summary. Clin. Infect. Dis..

[56] Liu J.C., Modha D.E., Gaillard E.A. (2013). What is the clinical significance of filamentous fungi positive sputum cultures in patients with cystic fibrosis?. J. Cyst. Fibros..

[57] Helmi M., Love R.B., Welter D., Cornwell R.D., Meyer K.C. (2003). Aspergillus Infection in Lung Transplant Recipients with Cystic Fibrosis: Risk factors and outcomes comparison to other types of transplant recipients. Chest.

[58] Herbrecht R., Denning D.W., Patterson T.F., Bennett J.E., Greene R.E., Oestmann J.-W., Kern W.V., Marr K.A., Ribaud P., Lortholary O. (2002). Voriconazole versus Amphotericin B for Primary Therapy of Invasive Aspergillosis. N. Engl. J. Med..

[59] Weigt S.S., Elashoff R.M., Huang C., Ardehali A., Gregson A.L., Kubak B., Fishbein M.C., Saggar R., Keane M.P., Lynch J.P. (2009). Aspergillus Colonization of the Lung Allograft Is a Risk Factor for Bronchiolitis Obliterans Syndrome. Am. J. Transplant..

[60] Colombo C., Burgel P.-R., Gartner S., van Koningsbruggen-Rietschel S., Naehrlich L., Sermet-Gaudelus I., Southern K.W. (2020). Impact of COVID-19 on people with cystic fibrosis. Lancet Respir. Med..

[61] Mathew H.R., Choi M.Y., Parkins M.D., Fritzler M.J. (2021). Systematic review: Cystic fibrosis in the SARS-CoV-2/COVID-19 pandemic. BMC Pulm. Med..

[62] Hofer M., Schmid C., Benden C., Speich R., Inci I., Weder W., Boehler A. (2012). Diabetes mellitus and survival in cystic fibrosis patients after lung transplantation. J. Cyst. Fibros..

[63] Spira A., Gutierrez C., Chaparro C., Hutcheon M.A., Chan C.K. (2000). Osteoporosis and Lung Transplantation: A prospective study. Chest.

[64] Gilljam M., Chaparro C., Tullis E., Chan C., Keshavjee S., Hutcheon M. (2003). GI Complications After Lung Transplantation in Patients with Cystic Fibrosis. Chest.

[65] Madill J., Gutierrez C., Grossman J., Allard J., Chan C., Hutcheon M., Keshavjee S.H. (2001). Nutritional assessment of the lung transplant patient: Body mass index as a predictor of 90–day mortality following transplantation. J. Heart Lung Transplant..

[66] Houwen R.H., van der Doef H.P., Sermet I., Munck A., Hauser B., Walkowiak J., Robberecht E., Colombo C., Sinaasappel M., Wilschanski M. (2010). Defining DIOS and Constipation in Cystic Fibrosis with a Multicentre Study on the Incidence, Characteristics, and Treatment of DIOS. J. Pediatr. Gastroenterol. Nutr..

[67] Morton J.R., Ansari N., Glanville A.R., Meagher A.P., Lord R.V.N. (2009). Distal Intestinal Obstruction Syndrome (DIOS) in Patients with Cystic Fibrosis After Lung Transplantation. J. Gastrointest. Surg..

[68] Dray X., Bienvenu T., Desmazes—Dufeu N., Dusser D., Marteau P., Hubert D. (2004). Distal intestinal obstruction syndrome in adults with cystic fibrosis. Clin. Gastroenterol. Hepatol..

[69] Green J., Gilchrist F.J., Carroll W. (2018). Interventions for preventing distal intestinal obstruction syndrome (DIOS) in cystic fibrosis. Cochrane Database Syst. Rev..

[70] Sayah D.M., Belperio J.A., Weigt S.S., Lynch J. (2015). Lung Transplantation for Cystic Fibrosis: Results, Indications, Complications, and Controversies. Semin. Respir. Crit. Care Med..

[71] Ramos K.J., Somayaji R., Lease E.D., Goss C.H., Aitken M.L. (2017). Cystic fibrosis physicians’ perspectives on the timing of referral for lung transplant evaluation: A survey of physicians in the United States. BMC Pulm. Med..

[72] Koutsokera A., Varughese R.A., Sykes J., Orchanian-Cheff A., Shah P.S., Chaparro C., Tullis E., Singer L., Stephenson A.L. (2019). Pre-transplant factors associated with mortality after lung transplantation in cystic fibrosis: A systematic review and meta-analysis. J. Cyst. Fibros..

[73] Upala S., Panichsillapakit T., Wijarnpreecha K., Jaruvongvanich V., Sanguankeo A. (2016). Underweight and obesity increase the risk of mortality after lung transplantation: A systematic review and meta-analysis. Transpl. Int..

[74] Morrell M.R., Pilewski J.M. (2016). Lung Transplantation for Cystic Fibrosis. Clin. Chest Med..

[75] Ramos K.J., Kapnadak S.G., Bradford M.C., Somayaji R., Morrell E.D., Pilewski J.M., Lease E.D., Mulligan M.S., Aitken M.L., Gries C.J. (2020). Underweight Patients with Cystic Fibrosis Have Acceptable Survival Following Lung Transplantation. Chest.

[76] Kelm D.J., Bonnes S.L., Jensen M.D., Eiken P.W., Hathcock M.A., Kremers W.K., Kennedy C.C. (2016). Pre-transplant wasting (as measured by muscle index) is a novel prognostic indicator in lung transplantation: A united network for organ sharing registry study. Clin. Transplant..

[77] Rozenberg D., Mathur S., Herridge M., Goldstein R., Schmidt H., Chowdhury N.A., Mendes P., Singer L.G. (2017). Thoracic muscle cross-sectional area is associated with hospital length of stay post lung transplantation: A retrospective cohort study. Transpl. Int..

[78] Jennerich A.L., Downey L., Goss C.H., Kapnadak S.G., Pryor J.B., Ramos K.J. (2023). Computed tomography body composition and clinical outcomes following lung transplantation in cystic fibrosis. BMC Pulm. Med..

[79] Thabut G., Mal H. (2017). Outcomes after lung transplantation. J. Thorac. Dis..

[80] Chan E.G., Hyzny E.J., Ryan J.P., Morrell M.R., Pilewski J., Sanchez P.G. (2022). Outcomes following lung re-transplantation in patients with cystic fibrosis. J. Cyst. Fibros..

[81] Iguidbashian J., Cotton J., King R.W., Carroll A.M., Gergen A.K., Meguid R.A., Fullerton D.A., Suarez-Pierre A. (2022). Survival following lung transplantation: A population-based nested case-control study. J. Card. Surg..

[82] Hayanga A.J., Du A.L., Joubert K., Tuft M., Baird R., Pilewski J., Morrell M., D’Cunha J., Shigemura N. (2018). Mechanical Ventilation and Extracorporeal Membrane Oxygenation as a Bridging Strategy to Lung Transplantation: Significant Gains in Survival. Am. J. Transplant..

[83] Halloran K., Aversa M., Tinckam K., Martinu T., Binnie M., Chaparro C., Chow C.-W., Waddell T., McRae K., Pierre A. (2018). Comprehensive outcomes after lung retransplantation: A single-center review. Clin. Transplant..

[84] Hall D.J., Belli E.V., Gregg J.A., Salgado J.C., Baz M.A., Staples E.D., Beaver T.M., Machuca T.N. (2017). Two Decades of Lung Retransplantation: A Single-Center Experience. Ann. Thorac. Surg..

[85] Ren D., Kaleekal T.S., Graviss E.A., Nguyen D.T., Sinha N., Goodarzi A., Agboli I., Suarez E.E., Loebe M., Scheinin S.A. (2018). Retransplantation Outcomes at a Large Lung Transplantation Program. Transplant. Direct.

[86] Iacono A.T., Johnson B.A., Grgurich W.F., Youssef J.G., Corcoran T.E., Seiler D.A., Dauber J.H., Smaldone G.C., Zeevi A., Yousem S.A. (2006). A Randomized Trial of Inhaled Cyclosporine in Lung-Transplant Recipients. N. Engl. J. Med..

[87] Iacono A., Wijesinha M., Rajagopal K., Murdock N., Timofte I., Griffith B., Terrin M. (2019). A randomised single-centre trial of inhaled liposomal cyclosporine for bronchiolitis obliterans syndrome post-lung transplantation. ERJ Open Res..

[88] Efficacy + Safety of Liposome Cyclosporine A to Treat Bronchiolitis Obliterans Post Single Lung Transplant (BOSTON-1)—Full Text View—ClinicalTrials.gov. https://clinicaltrials.gov/ct2/show/NCT03657342?term=inhaled+cyclosporine&cond=chronic+lung+transplant+rejection&draw=2&rank=1.

[89] Efficacy + Safety of Liposome Cyclosporine A to Treat Bronchiolitis Obliterans Post Single Lung Transplant (BOSTON-2)—Full Text View—ClinicalTrials.gov. https://clinicaltrials.gov/ct2/show/NCT03656926?term=inhaled+cyclosporine&cond=chronic+lung+transplant+rejection&draw=2&rank=2.

[90] Benden C., Goldfarb S.B., Stehlik J. (2019). An aging population of patients with cystic fibrosis undergoes lung transplantation: An analysis of the ISHLT Thoracic Transplant Registry. J. Heart Lung Transplant..

[91] Clausen E.S., Hadjiliadis D. (2021). Age at Lung Transplant Impacts Post-Transplant Survival in Cystic Fibrosis; Why?. Ann. Am. Thorac. Soc..

[92] Accurso F.J., Rowe S.M., Clancy J., Boyle M.P., Dunitz J.M., Durie P.R., Sagel S.D., Hornick D.B., Konstan M.W., Donaldson S.H. (2010). Effect of VX-770 in Persons with Cystic Fibrosis and the G551D-*CFTR* Mutation. N. Engl. J. Med..

[93] Fidler M.C., Beusmans J., Panorchan P., Van Goor F. (2017). Correlation of sweat chloride and percent predicted FEV1 in cystic fibrosis patients treated with ivacaftor. J. Cyst. Fibros..

[94] Ramos K.J., Pilewski J.M., Taylor-Cousar J.L. (2021). Challenges in the use of highly effective modulator treatment for cystic fibrosis. J. Cyst. Fibros..

[95] Benninger L.A., Trillo C., Lascano J. (2021). CFTR modulator use in post lung transplant recipients. J. Heart Lung Transplant..

[96] Smith M., Ryan K.J., Gutierrez H., Sanchez L.H.G., Anderson J.N., Acosta E.P., Benner K.W., Guimbellot J.S. (2022). Ivacaftor-elexacaftor-tezacaftor and tacrolimus combination in cystic fibrosis. J. Cyst. Fibros..

[97] Andersen D.H. (1938). Cystic fibrosis of the pancreas and its relation to celiac disease. Am. J. Dis. Child..

[98] Rommens J.M., Iannuzzi M.C., Kerem B., Drumm M.L., Melmer G., Dean M., Rozmahel R., Cole J.L., Kennedy D., Hidaka N. (1989). Identification of the cystic fibrosis gene: Chromosome walking and jumping. Science.

[99] Scott J., Hutter J., Stewart S., Higenbottam T., Hodson M., Penketh A., Wallwork J. (1988). Heart-lung transplantation for cystic fibrosis. Lancet.

